# *Ganoderma lucidum*—From Ancient Remedies to Modern Applications: Chemistry, Benefits, and Safety

**DOI:** 10.3390/antiox14050513

**Published:** 2025-04-25

**Authors:** Mădălina-Paula Plosca, Maria Simona Chiș, Anca Corina Fărcaș, Adriana Păucean

**Affiliations:** 1 Department of Food Engineering, Faculty of Food Science and Technology, University of Agricultural Sciences and Veterinary Medicine of Cluj-Napoca, 3–5 Manastur Street, 400372 Cluj-Napoca, Romania; madalina-paula.plosca@student.usamvcluj.ro (M.-P.P.); adriana.paucean@usamvcluj.ro (A.P.); 2 Department of Food Science, Faculty of Food Science and Technology, University of Agricultural Sciences and Veterinary Medicine of Cluj-Napoca, 3–5 Manastur Street, 400372 Cluj-Napoca, Romania; anca.farcas@usamvcluj.ro

**Keywords:** *Ganoderma lucidum*, bioactive compounds, anti-aging, anti-diabetic, anti-cancer, extraction methods

## Abstract

*Ganoderma lucidum* (*G. lucidum*), commonly known as Reishi or Lingzhi, is a medicinal mushroom with a rich history in traditional Asian medicine. This review examines diverse bioactive components supporting therapeutic properties, including polysaccharides, triterpenoids, phenolic compounds, fatty acids, peptides and proteins, vitamins, minerals, and sterols. The mushroom offers numerous health benefits, including immunomodulation, antioxidant and anti-inflammatory effects, liver protection, and anti-cancer activities. In addition, it shows potential in managing diabetes, cardiovascular disease, and viral infections. Advances in extraction technologies, such as ultrasound and microwave-assisted methods, have improved the bioavailability and efficacy of compounds. While *G. lucidum* is an excellent functional food and therapeutic agent it remains an unexploited source of nutrients. Further research is needed to optimize the industrial applications and evaluate the safety in specific populations.

## 1. Introduction

The stress and demands of modern life place significant strain on the immune system, which is particularly critical for those with weakened immunity, making them more vulnerable to infections and illness [1]. While technological advances have simplified many aspects of life, many people seek more effective natural alternatives. One such option is *Ganoderma lucidum* (*G. lucidum*)—a medicinal mushroom also known as “Reishi” and “Lingzhi”. *G. lucidum* has been valued for centuries, even millennia, for health-promoting properties associated with healing, longevity, wisdom, and happiness in China, Japan, and Asian countries [2,3,4].

It is a giant, dark mushroom with a shiny outer surface and a woody texture. The Latin term *lucidus* means “shiny” or “brilliant”, highlighting the mushroom’s varnished appearance. In China, this mushroom is known as lingzhi, while in Japan, members of the *Ganodermataceae* family are referred to as reishi or mannentake.

**Figure 1:**
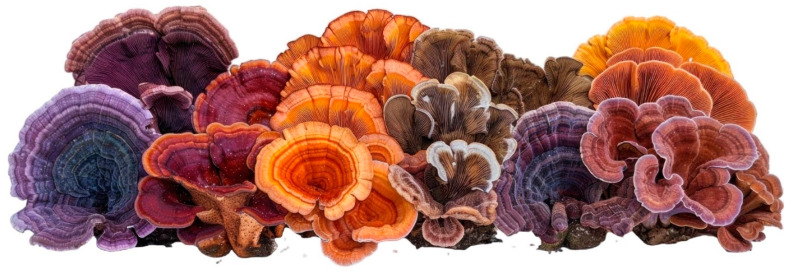
Color of *Ganoderma* species [8].

In total, 290 species within the family are classified under the genus *Ganoderma*. The basidiocarps of this genus feature a shiny surface due to thick-walled pilocystidia within an extracellular melanin matrix. *Ganoderma* species are distributed worldwide and are identified based on traits like the shape and color (red, black, blue/green, white, yellow, and purple) of the fruiting body, host specificity, and geographic origin [3]. The diverse species of *G. lucidum* mushrooms and their colors and structures are illustrated in Figure 1 and Table 1.

**Table 1:** Color, characteristics, and medicinal uses of *Ganoderma* species [5,6,7].

No	Color /Taste	Name of Species	Common Name	Medicinal Uses
1.	Black/Salty	*Ganoderma neojaponicum*	Imazeki or Black lingshi	Improves lung function, ethnomedicinal potential, cytotoxic effects
2.	White/Hot	*Ganoderma applanatum*	Bear bread or artist’s bread	Protects kidney; skin-whitening and anti-wrinkle potential (anti-tyrosinase, anti-collagenase, anti-elastase).
3.	Red/Bitter	*Ganoderma lucidum*	Lingzhi or Reishi	Acids internal organs and improve memory
4.	Yellow/Sweet	*Ganoderma Curtisii*	Golden reishi	Strengthens spleen function
5.	Purple/Violaceous/Sweet	*Ganoderma sinense*	Zizhi	It enhances the function of eyes, and joints, and helps the complexion

However, these morphological characteristics can vary due to differences in cultivation conditions, climate, and natural genetic variations, leading to numerous synonyms and a complex, overlapping taxonomy. Some taxonomists argue that macromorphological features have limited value in identifying *Ganoderma* species because of their high phenotypic plasticity. More reliable identification methods include examining spore shape and size, context color and consistency, and the microanatomy of the pillar crust [3,9,10].

*G. lucidum* has been regarded as a medicinal mushroom for over two millennia, and its benefits have been noted in early Chinese writings. One of the oldest pharmacological texts, the *Shen Nong Ben Cao Jing* from the Eastern Han dynasty, classified *G. lucidum* as a top-grade, non-toxic remedy. Further texts, like the *Compendium of Materia Medica*, outlined its properties for enhancing energy, memory, and overall vitality, attributing it to anti-aging effects [11]. The mushroom was featured in Taoist-inspired art and appeared in paintings, carvings, and accessories. Various ancient texts describe *G. lucidum* growing on decaying wood or soil, with different colors symbolizing unique qualities, such as a red color resembling coral or a black-like lacquer. Its preparation was believed to promote clarity, calm the mind, and support kidney function. Once rare and primarily accessible to the wealthy, *G. lucidum* was associated with the homes of immortals, its scarcity and mystique reinforcing its reputation as a powerful tonic. Today, *Ganoderma* mushrooms remain essential in traditional Asian medicine, and their global popularity continues to grow [12,13].

Medicinal mushrooms have drawn significant research attention, particularly for their biologically active compounds, including polysaccharides like β-glucans, polysaccharide-peptides, polysaccharide-protein complexes, terpenoids, sterols, and phenolic compounds [14]. These substances have been investigated for their anti-cancer, antioxidant, anti-inflammatory, and immunomodulatory potential effects [1,15,16]. Research consistently demonstrates that *G. lucidum* boosts immune function and reduces oxidative stress, which may, in turn, improve productivity in livestock and poultry. Polysaccharides from *G. lucidum* (GLP) are considered one of the mushroom’s primary bioactive compounds and are widely used as health supplements [17]. Additionally, GLP has been shown to interact with and regulate gut microbiota, indicating its potential to support intestinal health [4,18].

Therefore, the present manuscript is an up-to-date review of *G. lucidum* bioactive compounds extraction methods and its chemical composition. Moreover, therapeutic, pharmaceutical, cosmetic and its food applications are highlighted together with its safety evaluation.

## 2. Extraction

**Figure 2:**
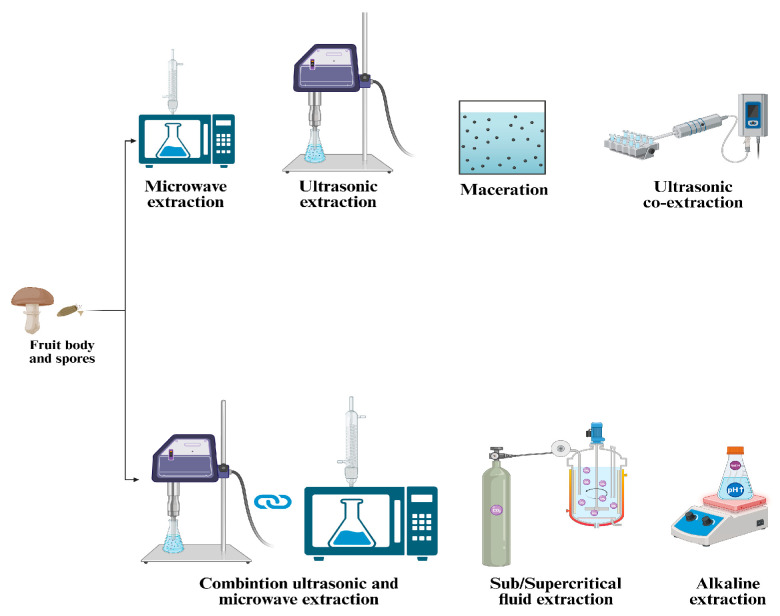
Overview of extraction techniques applied to *Ganoderma lucidum*.

*G. lucidum* has a fragile outer shell that must be broken to release its bioactive content. Breaking the wall enhances oil extraction efficiency and facilitates the bioavailability and absorption of bioactive compounds in the human body. The extraction process is essential for isolating the bioactive compounds of *G. lucidum*, ensuring both their therapeutic effectiveness and commercial viability. The choice of extraction technique significantly affects the yield, purity, and bioactivity of the resulting compounds. Key methods for extracting bioactive compounds from *G. lucidum* include ethanol precipitation, microwave-assisted extraction (MAE), ultrasonic-assisted co-extraction (UACE), supercritical CO_2_ extraction, and other methods illustrated in Table 2 and Figure 2 [19].

Zheng et al. (2020) explored the extraction of polysaccharides and triterpenoids through hot water extraction, ethanol precipitation, and ultrasonic-assisted co-extraction (UACE). Their findings revealed that extracts obtained with UACE exhibited superior antioxidant activity [20]. In a study conducted by Do et al. (2021), the extraction of polysaccharides involved a combination of ultrasonic-assisted extraction and enzymatic extraction methods. The process was further optimized using response surface methodology to maximize the yield of polysaccharides [21]. Huang et al. (2010) analyzed the MAE in a study that combined the ultrasonic-assisted extraction (UAE) and MAE methods (UMAE) and compared them to hot water extraction and UAE. The results demonstrated that the UMAE method produced a higher yield of polysaccharides than the traditional methods [22].

The supercritical CO_2_ method is commonly used for spore oil extraction because it produces high oil yields, requires little energy, and prevents spore oil oxidation.

Innovative technologies are being refined to efficiently extract bioactive compounds from *G. lucidum* while reducing environmental and health concerns. Techniques such as microwave-assisted, ultrasonic-assisted, pressurized, and supercritical fluids achieve high yields with minimal or no organic solvents. Extraction efficiency depends on the process duration, temperature, solvent-to-sample ratio, extraction cycles, and type of solvent. Higher temperatures enhance solubility, accelerating extraction by reducing solvent viscosity and surface tension. Additional purification steps may be required to eliminate impurities like waxes, chlorophylls, and lipids [23].

Many researchers are currently exploring the combined application of innovative extraction technologies. However, these methods require further refinement and optimizing the conditions is essential to enable their successful scalability for industrial processes [24]. The main extraction methods used for *G. lucidum* bioactive compounds are illustrated in Figure 2.

**Table 2:** Extraction methods from *G. lucidum* to obtain bioactive compounds.

Source	Extraction Methods	Parameters	Bioactive Compound (Yields)	References
*Ganoderma lucidum* fruiting bodies	Microwave-assisted extraction	300–600 W, 70% ethanol, 10–30 min	Polysaccharides: 13.08% Triterpenoids: 9.15%	[25]
*Ganoderma lucidum* mycelium	Ultrasonic-assisted extraction	140–245 W, 50% ethanol, 30 min	Polysaccharides: 6.1% Phenolic compounds: 1.8%	[26]
*Ganoderma lucidum* fruiting bodies	Hot water extraction/ethanol maceration	70–100 °C, 2–6 h, 96% ethanol	Polysaccharides: 4–8% Triterpenoids: 1.2–1.5%	[20,27]
*Ganoderma lucidum* spores	Ultrasonic/Microwave-assisted extraction	50 W, 40 kHz, 284 W, Water, 11.7 min	Polysaccharides: 3.27%	[22]
*Ganoderma lucidum* fruiting bodies	Ultrasonic-assisted co-extraction	210 W, 40 kHz, 50% ethanol, 25 min	Polysaccharides: 6.0% Triperpenoids: 2.5%	[20]
*Ganoderma lucidum* fruiting bodies	Supercritical CO_2_ extraction	43 MPa, 54.8 °C CO_2_: 7 mL/min	Triterpenoids: 1.56 mg/100 g	[28]
*Ganoderma lucidum* fruiting bodies	Alkaline extraction	60 °C, 77.3 min, 5.1% NaOH	Polysaccharides: 8.2%	[29]
*Ganoderma lucidum* fruiting bodies	Subcritical water extraction	180 °C, 7 MPa, 1 mL/min	Triterpenoids and Phenolic compounds: 58.42%	[30]

## 3. Bioactive Compounds

Like many mushrooms, *G. lucidum* consists of about 90% water by weight, while the remaining 10% comprises various nutritional and bioactive components, including polysaccharides, triterpenoids, sterols, phenolic compounds, and proteins. Studies indicate that the mushroom contains 26–28% carbohydrates, 3–5% crude fat, 59% fiber, 7–8% protein, and 1.8% ash in its dry form. Additionally, *G. lucidum* is rich in essential minerals such as potassium, phosphorus, calcium, magnesium, selenium, zinc, and iron, contributing to its health-promoting properties [3,14,31].

Among its bioactive components, polysaccharides are the most abundant, with structural variations influencing their biological activity. Triterpenoids, including ganoderic acids, are another prominent group recognized for their structural complexity and functional diversity. The mushroom’s phenolic compounds, sterols, and other nutrients complement its bioactive profile, making it a subject of extensive research. This unique composition supports traditional uses and highlights its potential in modern medicinal and nutritional applications [3,32].

### 3.1. Polysaccharides

Mushrooms, including *G. lucidum*, are notable for their high content of polysaccharides, which are structurally diverse macromolecules with significant biological functions. These polysaccharides, extracted from the mushroom’s fruiting body, spores, and mycelia, are composed of β-glucans, particularly β-1,3-D-glucopyranan backbones with β-1,6-linked monoglucosyl side chains [33,34]. Such structural configurations are critical for interacting with immune system receptors, influencing bioactivity. *G. lucidum* polysaccharides also include heteropolysaccharides, which combine multiple monosaccharides like glucose, mannose, and galactose, enhancing their functional diversity [35].

The molecular weight and tertiary structures, such as the triple helix conformation, significantly affect the biological activity of these compounds. Polysaccharides with higher molecular weights are often associated with more significant immunomodulatory activity, though variations in branching can influence water solubility and receptor recognition [33,35].

### 3.2. Triterpenoids

Triterpenoids in *G. lucidum*, particularly ganoderic acids, are a class of lanostane-type compounds characterized by their complex tetracyclic structure. These molecules often contain hydroxyl (-OH), carboxyl (-COOH), or keto (=O) groups, which contribute to their diverse functionality [36,37].

Over 380 different triterpenoids, including lucidenic acids and ganodermanontriol, have been identified, each with structural variations that affect solubility and bioactivity [38].

These triterpenoids are typically concentrated in the mushroom’s fruiting body and spores. They are often extracted using ethanol or supercritical CO_2_, followed by chromatographic purification [39,40]. The lanostane backbone and associated functional groups are essential for their biochemical interactions. Recent research highlights the role of these triterpenoids in maintaining fungal cell wall integrity and regulating internal fungal metabolism [36,37,39,41].

### 3.3. Phenolic Compounds

The phenolic compounds in *G. lucidum* include cinnamic acid derivatives, benzoic acids, and flavonoids [42]. These compounds are characterized by their aromatic ring structures with attached hydroxyl groups, which provide their antioxidant capabilities. Phenolics are primarily found in the fruiting body and spores, where they serve as protective agents against oxidative stress and microbial attack [43].

Common phenolic acids in *G. lucidum* include caffeic, ferulic, and gallic acids [42,44]. These compounds can be extracted using ethanol or aqueous methanol solutions, and their purity is often analyzed using high-performance liquid chromatography (HPLC) [45]. The complexity of their aromatic structures and hydroxyl substitutions significantly influences their ability to interact with reactive oxygen species [43].

### 3.4. Sterols

Sterols in *G. lucidum*, such as ergosterol and ergosta-5,7,22-trien-3β-ol, are essential components of the fungal cell membrane. These molecules are precursors to vitamin D2 and play a role in maintaining membrane fluidity [46]. Ergosterol, in particular, is abundant and often serves as a marker for the quality of *G. lucidum* extracts.

Ergosterol has been extensively studied for its structural integrity under UV exposure, leading to its conversion into vitamin D2 [47]. Sterols are generally extracted using nonpolar solvents such as hexane or supercritical CO_2_ [48].

### 3.5. Proteins and Peptides

*G. lucidum* is a rich source of bioactive proteins and peptides that contribute significantly to its therapeutic profile. The most notable is Ling Zhi-8 (LZ-8), a fungal protein extracted from the mushroom’s mycelium and fruiting body. It comprises 110 amino acids and forms a biologically active homodimer with an immunoglobulin-like, non-covalently linked structure. This structure plays a role in immunomodulation and tumor growth inhibition [49]. It is extracted using saline solutions and purified through ion-exchange chromatography. The glycan portion of LZ-8, primarily composed of mannose, galactose, and glucose, plays a critical role in its interactions with biological systems, influencing immune-regulatory and anti-inflammatory activities [50,51].

Additionally, *G. lucidum* produces bioactive peptides, typically 5–21 amino acids, exhibiting antioxidative, antimicrobial, and immunomodulatory properties. For example, antimicrobial peptides are composed of hydrophobic amino acids, β-sheets, α-helices, random coil structures, and disulfide bonds [52]. The proteins and peptides in *G. lucidum* collectively play a synergistic role in the mushroom’s health-promoting properties, forming a robust biochemical framework for therapeutic applications.

### 3.6. Minerals

*G. lucidum* contains a rich array of minerals, which are inorganic elements that play essential roles in physiological processes. These minerals are necessary for various biological functions, from enzyme activation to maintaining cellular structure and function. The key minerals found in *G. lucidum* include the following [53]:

- Potassium (K): An essential mineral that is critical for maintaining the balance of fluids within cells and tissues, as well as for nerve transmission and muscle contraction. Potassium is crucial for maintaining fluid balance, nerve transmission, and muscle contraction. *G. lucidum* contains approximately 432 mg of potassium per 100 g of the mushroom [53,54].
- Calcium (Ca): A vital element for the formation of bone and teeth, muscle contraction, and nerve signaling. It also plays a crucial role in the release of hormones and enzymes. The mushroom provides about 1.88 mg of calcium per 100 g [53,54].
- Magnesium (Mg): An essential mineral in immune function, influencing processes such as immune cell adhesion, the production of immunoglobulins, the interaction between lymphocytes and Immunoglobulin M (IgM), antibody-mediated cytolysis, and the response of macrophages to lymphokines. *G. lucidum* contains 7.95 mg of magnesium per 100 g [53,54].
- Iron (Fe): A critical component of hemoglobin, the protein responsible for wearing oxygen in the blood. Iron is also involved in cell energy production and supports overall metabolic processes. The mushroom offers 2.22 mg of iron per 100 g [53,54].
- Zinc (Zn): A trace element vital for immune system function, DNA synthesis, protein synthesis, and cell division. Zinc also contributes to wound healing and maintaining skin and hair health. *G. lucidum* provides 0.7 mg of zinc per 100 g [53,54].
- Manganese (Mn): A trace mineral involved in forming connective tissue, bone health, and wound healing. Manganese also acts as a cofactor for various enzymes, including those involved in antioxidant defense. The mushroom contains 22 mg of manganese per 100 g [53,54].
- Phosphorus (P): Essential for repairing cells and tissues and growing the protein. *G. lucidum* contains 225 mg of phosphorus per 100 g [53,54].
- Sulfur (S): Essential for immune function and blood clotting and is responsible for transport across cell membranes. The mushroom provides 129 mg of sulfur per 100 g [53,54].
- Sodium (Na): Helps maintain electrolytic balance and nerve function. *G. lucidum* contains 2.82 mg of sodium per 100 g [53,54].
- Copper (Cu): Involved in iron metabolism and red blood cell formation. The mushroom provides 26 mg of copper per 100 g [53,54].

The mineral content of *G. lucidum* is not fixed and can vary significantly based on environmental factors, cultivation practices, and post-harvest processing. For instance, Ogbe and Obeka reported variations in calcium (1.99%), magnesium (0.34%), potassium (1.11%), sodium (229.88 ppm), zinc (51.49 ppm), phosphorus (30.17 ppm), manganese (71.06 ppm), copper (7.43 ppm), and iron (121.37 ppm) in wild specimens collected in Nigeria, attributing these differences to the type of substrate and soil composition [55]. Senila et al. compared their study with the literature and demonstrated that local climatic conditions and growth stages influence mineral profiles [56]. Additional research by Akinyeye et al. and Muhammad et al. highlighted the importance of iron and zinc in supporting health and performance, while noting that antinutrients like phytates could reduce their bioavailability [57,58]. Thus, this mushroom’s mineral profile reflects a complex interaction between environmental factors, processing techniques, and the specific analytical methods employed.

### 3.7. Lipids and Fatty Acids

*G. lucidum* contains a variety of lipids and fatty acids, which are essential components of its bioactive profile. Lipids serve as energy reserves, structural components of cell membranes, and precursors to signaling molecules. The mushroom lipid composition includes sterols, phospholipids, and neutral lipids [59,60].

Fatty acids in *G. lucidum* are both saturated and unsaturated. Unsaturated fatty acids, such as oleic acid, linoleic acid, and palmitoleic acid, are particularly abundant. These acids play critical roles in maintaining cellular membrane integrity and fluidity. The mushroom polyunsaturated fatty acids (PUFAs) contribute to its biochemical and nutritional complexity. Saturated fatty acids, such as palmitic acid, are also detected, albeit in smaller amounts. These lipids and fatty acids enrich the overall bioactive composition of *G. lucidum* [60,61].

### 3.8. Vitamins

*G. lucidum* is a natural source of several vitamins and essential organic compounds required for metabolic processes. These vitamins enhance the mushroom’s nutritional value and contribute to its role as a functional food [54,62].

Vitamin B Complex: *G. lucidum* contains a range of B vitamins, including the following [63]:

- Vitamin B1 (Thiamine): Plays a role in nerve function and energy production [64]. *G. lucidum* contains 1.14 mg/100 g. The recommended daily intake (DRI) for Vitamin B1 is 1.0 mg [65].
- Vitamin B2 (Riboflavin): Involved in energy metabolism and supports maintaining healthy skin and eyes [64]. *G. lucidum* contains 1.86 mg/100 g. The DRI for Vitamin B2 is 1.1 mg [65].
- Vitamin B3 (Niacin): Aids in DNA repair and energy production through redox reactions [64]. *G. lucidum* contains 21.42 mg/100 g. The DRI for Vitamin B3 is 12 mg [65].
- Vitamin B9 (Folate): Essential for DNA synthesis and cell production [66]. *G. lucidum* contains 287.45 µg/100 g. The DRI for Vitamin B9 is 320 µg [65].

*G. lucidum* contains Vitamin D [54], primarily in the form of ergosterol (a precursor to vitamin D2). This vitamin supports calcium absorption and bone health and is synthesized when ergosterol is exposed to sunlight or UV light [67].

Vitamin C (Ascorbic Acid) [54]: Though present in small amounts (2.98 mg/100 g), vitamin C is an antioxidant and plays a role in collagen synthesis and immune function [65,68].

Vitamin E (Tocopherol) [54]: *G. lucidum* contains tocopherols (0.36 mg/100 g), lipid-soluble antioxidants that protect cellular membranes from oxidative damage [65,69,70].

## 4. Applications of *G. lucidum*

**Figure 3:**
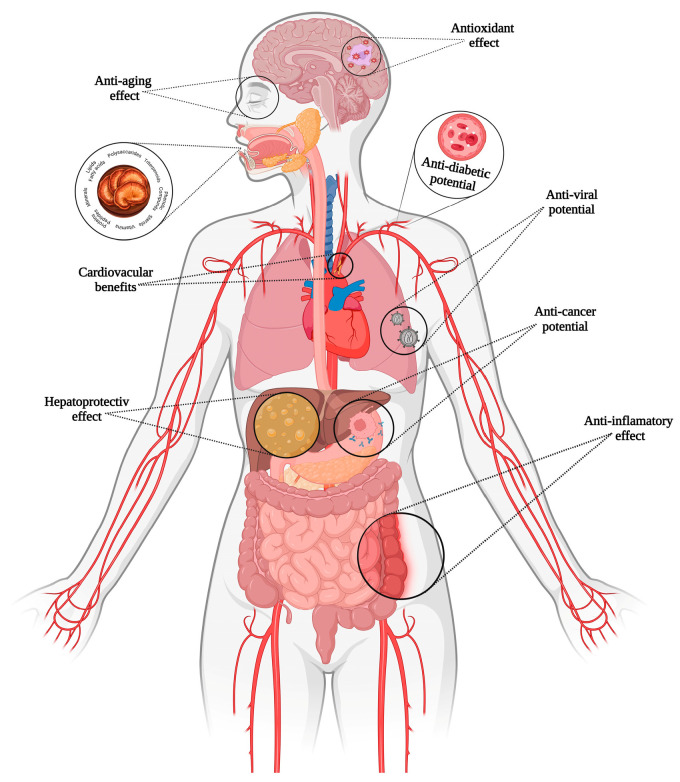
Benefits of *Ganoderma lucidum* bioactive compounds.

*G. lucidum*’s nickname, “the mushroom of immortality”, reflects its reputation for promoting longevity and vitality. Revered initially in China and Japan, *G. lucidum* has become a global symbol of natural health, backed by increasing scientific evidence. Its bioactive components, including triterpenoids, polysaccharides, and peptides, make it an essential of modern integrative [71]. Different applications of *G. lucidum* are illustrated in Figure 3.

### 4.1. Therapeutic Applications

#### 4.1.1. Immunomodulatory Properties

One of the most well-documented properties of *G. lucidum* is its ability to modulate the immune system. This dual action allows it to enhance a weakened immune response or suppress an overactive one, making it invaluable for various conditions, from infections to autoimmune diseases and cancer.

Polysaccharides, particularly β-glucans, are central to *G. lucidum* immunomodulatory effects. These compounds bind to immune cell receptors such as Dectin-1 and Toll-like receptors (TLRs), triggering pathways that activate macrophages, dendritic cells, and neutrophils. For example, Dectin-1 in different cells produces signal transduction and contributes to immune response. Similarly, TLR activation promotes the release of cytokines that prime the immune system to respond to pathogens [72].

Natural killer (NK) cells and cytotoxic T-cells, which are crucial for targeting virus-infected and cancerous cells, are also stimulated by *G. lucidum*. The mushroom enhances its cytotoxic activity by increasing the production of interferon-gamma (IFN-γ), a cytokine that strengthens immune responses. Clinical studies have shown that cancer patients who consumed *G. lucidum* experienced significant improvements in NK cell function and T-cell proliferation, demonstrating its role as an adjunct therapy in oncology [72].

*G. lucidum* modulates cytokine production to maintain immune equilibrium. It suppresses pro-inflammatory cytokines like tumor necrosis factor-alpha (TNF-α) and interleukin-6 (IL-6), often elevated in chronic inflammatory and autoimmune conditions, while enhancing anti-inflammatory cytokines such as IL-10 and TGF-β. This balance helps prevent immune system overactivation, reducing tissue damage [72,73].

Studies examined the effects of *G. lucidum* on patients with advanced-stage cancer. The results indicated an enhancement in immune responses, with increased activity of NK cells and improved quality of life among participants. However, the study also noted that while *G. lucidum* could be beneficial as an adjunct therapy, it should not replace conventional cancer treatments [74,75].

#### 4.1.2. Anti-Inflammatory Properties

Chronic inflammation is a hallmark of many modern diseases, including cardiovascular disorders, diabetes, and neurodegenerative conditions. *G. lucidum* contains triterpenoids, potent bioactive compounds that inhibit key inflammatory pathways, making it a valuable anti-inflammatory agent [76].

One of the primary mechanisms involves the suppression of the nuclear factor-kappa B (NF-κB) signaling pathway. NF-κB is a factor that regulates the production of pro-inflammatory cytokines. By inhibiting this pathway, *G. lucidum* reduces the production of cytokines like TNF-α, IL-1β, and IL-6, alleviating chronic inflammation [77].

*G. lucidum* reduces the synthesis of prostaglandins and leukotrienes, inflammatory mediators derived from arachidonic acid. This effect is particularly beneficial in conditions like arthritis, where these molecules drive joint inflammation and pain [78]. In clinical settings, patients with rheumatoid arthritis reported significant reductions in pain and swelling after three months of supplementation with *G. lucidum* [79].

Its anti-inflammatory properties also extend to the nervous system. By modulating neuroinflammatory pathways, *G. lucidum* may protect against conditions such as Alzheimer’s disease and multiple sclerosis, where chronic inflammation contributes to disease progression [79].

#### 4.1.3. Antioxidant Properties

Oxidative stress, caused by excess free radicals, is a significant factor in aging and the development of chronic diseases. *G. lucidum* is a rich source of antioxidants, including phenolic compounds, polysaccharides, and triterpenoids, which protect cells from oxidative damage.

The mushroom scavenges free radicals directly and enhances the body’s endogenous antioxidant defenses. It increases the activity of enzymes like superoxide dismutase (SOD), catalase, and glutathione peroxidase, which neutralize harmful reactive oxygen species (ROS). This dual action prevents oxidative damage to lipids, proteins, and DNA, reducing the risk of conditions like cancer, cardiovascular disease, and neurodegeneration [15,80].

Studies have shown that elderly individuals who consumed *G. lucidum* experienced significant reductions in oxidative stress markers and improved antioxidant enzyme activity. These findings highlight its potential as an anti-aging agent and a preventive measure against oxidative damage-related diseases [15,17,80].

#### 4.1.4. Hepatoprotective Effects

As the body’s primary detoxification organ, the liver is vulnerable to damage from toxins, infections, and oxidative stress. *G. lucidum* offers robust hepatoprotective effects, primarily through its triterpenoid content [81].

These compounds prevent lipid peroxidation in liver cells, a process that can lead to cell membrane damage and liver dysfunction [81]. Additionally, *G. lucidum* enhances the liver’s detoxification capacity by increasing the activity of enzymes, which neutralize harmful metabolites [82].

In patients with non-alcoholic fatty liver disease (NAFLD), supplementation with *G. lucidum* improved liver enzyme profiles and reduced fat accumulation [83]. Similarly, patients with chronic hepatitis experienced reduced fibrosis and better liver function, demonstrating its potential in managing both metabolic and infectious liver conditions [41,82,84,85,86].

#### 4.1.5. Anti-Cancer Potential

The anti-cancer properties of *G. lucidum* are among its most extensively studied benefits. Its bioactive compounds target cancer cells through multiple mechanisms while sparing healthy cells. A primary mechanism is the induction of apoptosis, or programmed cell death, in cancer cells. *G. lucidum* achieves this by activating caspases and disrupting mitochondrial function. Additionally, it inhibits angiogenesis, forming new blood vessels that supply tumors, thereby restricting tumor growth and metastasis [35].

The mushroom modulates the immune system to enhance the body’s natural anti-cancer defenses. *G. lucidum* strengthens the immune system’s ability to recognize and eliminate cancer cells by boosting NK cell activity and increasing T-cell-mediated responses [87,88].

Various studies have analyzed *G. lucidum* polysaccharides, which exhibited anti-cancer effects through cytotoxicity, antioxidative properties, and apoptosis induction.

Zhong et al. carried out an in vitro investigation using GLP at concentrations ranging from 0 to 15 mg/mL to explore their anti-cancer properties via cytotoxicity mechanisms [89].

YouGuo et al. examined the antioxidant effects of GLP, administering doses of 100–300 mg/kg twice daily to rats with ovarian cancer [90].

Jin et al. studied the impact of GLP on cervical cancer cells in vitro, focusing on its ability to induce apoptosis. Cancer cells were treated with GLP at concentrations of 0–500 μg/mL for a duration of 72 h [91].

YouGuo et al. demonstrated that *G. lucidum* polysaccharides reduce oxidative stress by lowering malondialdehyde levels and enhancing antioxidant enzyme activity in ovarian cancer-induced rats. Jin et al. showed that these polysaccharides induce apoptosis, inhibit migration and invasion of cervical cancer cells (C-33A and HeLa), and block EMT and JAK/STAT5 signaling pathways. Zhong et al. highlighted their ability to reduce tumor malignancy and disrupt autophagic flux in various in vitro and in vivo models, confirming the broad therapeutic potential of *G. lucidum* polysaccharides in oncology [89,90,91].

### 4.2. Pharmaceutical Applications

*G. lucidum*, widely recognized for its broad therapeutic properties, has shown considerable promise in several pharmaceutical domains, particularly in managing chronic diseases such as diabetes, cardiovascular disorders, and viral infections. Its bioactive compounds, such as polysaccharides and triterpenoids, play critical roles in regulating metabolic pathways, immune function, and cellular processes, making it a valuable candidate in modern medicine [92].

#### 4.2.1. Anti-Diabetic Potential

Diabetes, particularly type 2 diabetes, is a global health crisis characterized by insulin resistance and elevated blood sugar levels. *G. lucidum* has emerged as a promising natural remedy for managing diabetes due to its ability to regulate glucose metabolism and enhance insulin sensitivity [93].

Polysaccharides are the primary bioactive components responsible for *G. lucidum* anti-diabetic effects, which enhance the body’s ability to metabolize glucose. Studies have demonstrated that *G. lucidum* can activate the pathway of AMP-activated protein kinase (AMPK), which is key in improving insulin sensitivity and cell glucose uptake. This mechanism is particularly beneficial in type 2 diabetes, where insulin resistance is a primary concern [94,95].

*G. lucidum* also helps regulate blood sugar levels by inhibiting the action of alpha-glucosidase, an enzyme responsible for breaking down carbohydrates into glucose in the small intestine with an insulin sensitivity effect. By inhibiting this enzyme, *G. lucidum* reduces the rate at which carbohydrates are digested, leading to slower glucose absorption and more stable blood sugar levels post-meal [93].

Clinical trials and animal studies have shown promising results in diabetic patients supplemented with *G. lucidum*. In one study, patients who took *G. lucidum* extract experienced a significant reduction in fasting blood glucose levels and hemoglobin A1c (HbA1c), a marker of long-term blood sugar control. This demonstrates the potential of *G. lucidum* as an adjunct treatment for managing diabetes, alone or alongside conventional therapies [94,96].

#### 4.2.2. Cardiovascular Benefits

Cardiovascular diseases, including hypertension, atherosclerosis, and ischemic heart disease, are leading causes of mortality worldwide. *G. lucidum* has been shown to offer significant cardiovascular benefits through various mechanisms that support heart health and reduce common cardiovascular risk factors [82].

One of the most notable benefits is the ability to regulate blood pressure. *G. lucidum* contains triterpenoids that act as natural angiotensin-converting enzyme (ACE) inhibitors, which help relax blood vessels and lower blood pressure [97]. By inhibiting the ACE, *G. lucidum* reduces the production of angiotensin II, a peptide that constricts blood vessels and raises blood pressure. This effect is particularly beneficial for individuals with hypertension, as it can help normalize blood pressure levels [98].

Additionally, *G. lucidum* anti-inflammatory and antioxidant properties play a crucial role in preventing the development of atherosclerosis, a condition in which fatty deposits accumulate in the arteries. By reducing oxidative stress and inflammatory markers, *G. lucidum* protects endothelial cells (the cells lining the blood vessels) from damage, preventing plaque formation and promoting healthy blood flow [99,100,101].

Furthermore, *G. lucidum* has been shown to improve lipid profiles by reducing low-density lipoprotein (LDL) cholesterol and increasing high-density lipoprotein (HDL) cholesterol. In a clinical trial involving individuals with hyperlipidemia, supplementation with *G. lucidum* extract significantly reduced LDL cholesterol levels, further supporting its role in managing cardiovascular health [102].

#### 4.2.3. Anti-Viral Properties

*G. lucidum* has long been recognized for its immune-enhancing properties, and emerging research has highlighted the potential in fighting viral infections. The mushroom anti-viral effects are attributed mainly to its polysaccharides and triterpenoids, which activate immune cells and interfere with viral replication [72].

One of the key mechanisms through which *G. lucidum* combats viral infections is stimulating NK cells and enhancing the body IFN-γ production. These immune responses are crucial for recognizing and eliminating virus-infected cells. The ability of *G. lucidum* to boost NK cell activity increases the immune system ability to fight off viruses such as the herpes simplex virus (HSV), influenza, and hepatitis B [103].

Additionally, *G. lucidum* has demonstrated direct anti-viral effects by inhibiting the replication of viruses. For example, studies have shown that *G. lucidum* extract can inhibit the replication of the human immunodeficiency virus (HIV) by preventing the virus from binding to immune cells [104]. Similarly, it has been shown to block the replication of influenza viruses by interfering with viral protein synthesis, providing a natural alternative to conventional anti-viral treatments [105].

These findings make *G. lucidum* a promising candidate for enhancing immune defense against a wide range of viral infections, particularly in individuals with weakened immune systems or those at high risk of viral exposure.

### 4.3. Cosmetic Applications

*G. lucidum*’s beneficial effects are not limited to internal health but also include skincare and cosmetic applications. Its antioxidant, anti-inflammatory, and anti-aging properties have made it a highly sought-after ingredient in various cosmetic formulations. From reducing wrinkles to brightening the complexion, *G. lucidum* is a powerful ally in the fight against skin aging and damage [82].

#### 4.3.1. Anti-Aging Effect

As the skin ages, its ability to repair itself diminishes, leading to wrinkles, fine lines, and sagging. *G. lucidum’s* antioxidant properties play a crucial role in combating the visible signs of aging by neutralizing free radicals that accelerate skin aging. The phenolic compounds and triterpenoids in *G. lucidum* protect skin cells from oxidative damage caused by environmental stressors like UV radiation and pollution [106,107].

*G. lucidum* also supports collagen synthesis, a critical factor in maintaining skin elasticity and firmness [108]. Promoting the production of collagen and elastin helps reduce the appearance of wrinkles and sagging, giving the skin a firmer and more youthful appearance. Clinical studies have demonstrated that regular use of *G. lucidum* extract in cosmetic products can significantly improve skin texture and elasticity, providing visible anti-aging benefits [107].

#### 4.3.2. Skin Brightening and Reassuring Properties

For many, hyperpigmentation and uneven skin tone are persistent concerns, commonly caused by sun exposure, aging, or hormonal imbalances. *G. lucidum* effectively tackles these issues by regulating melanin production. The triterpenoids in this medicinal mushroom inhibit tyrosinase activity, an enzyme crucial to melanin synthesis. By reducing melanin production, *G. lucidum* minimizes the appearance of dark spots, age spots, and other forms of discoloration, leading to a more radiant and even skin tone [109]. Clinical studies have demonstrated that skincare products infused with *G. lucidum* can significantly enhance skin brightness and reduce pigmentation, offering a natural and safe alternative to synthetic brightening agents while supporting overall skin health [106].

In addition to its brightening properties, *G. lucidum* excels in its anti-inflammatory and soothing capabilities, making it ideal for managing inflammatory skin conditions such as acne, eczema, and psoriasis. These conditions are often exacerbated by the overproduction of inflammatory cytokines like TNF-α and IL-1β, which contribute to redness, swelling, and discomfort. *G. lucidum* triterpenoids effectively suppress these cytokines, calming inflammation and promoting comfort for irritated skin [109].

Moreover, *G. lucidum* is renowned for accelerating wound healing and supporting tissue repair. It enhances the skin’s natural regenerative processes, aiding in recovering scars, minor injuries, and inflamed areas. Soothing the skin and reducing redness helps restore a balanced complexion and prevents recurring irritation. At the same time, its regenerative properties promote the repair of damaged tissues and reinforce the skin’s resilience over time [109,110].

By addressing multiple skin concerns, *G. lucidum* offers an extensive approach to achieving healthier, more radiant skin. Whether incorporated into creams, serums, or masks, this medicinal mushroom is a cornerstone of modern skincare, providing immediate and long-term benefits for skin health and appearance.

### 4.4. Food Applications

To provide functional health benefits, *G. lucidum* is increasingly included in various food products, such as teas, coffees, smoothies, and energy bars. Its high concentration of polysaccharides and antioxidants makes it an ideal addition to functional foods, offering enhanced immune support, stress relief, and antioxidant protection [54].

Studies have shown that incorporating *G. lucidum* into functional foods can improve gut health by promoting beneficial gut bacteria, improving digestion, and supporting overall metabolic function. Additionally, regular consumption of *G. lucidum* in food form has been associated with enhanced mental clarity, better mood regulation, and improved cognitive function, making it a valuable ingredient in brain-boosting supplements [32,99].

## 5. Safety Evaluation

Modern scientific evaluations confirm that *G. lucidum* is safe for therapeutic, cosmetic, and dietary applications, provided it is consumed within recommended guidelines. It is categorized as a Class 1 substance in the Plant Safety Manual published by the American Herbal Products Association [12]. In the United States, extracts such as beta-glucans from the mycelium of *G. lucidum* have received GRAS (*Generally Recognized As Safe*) status, allowing their use in food products such as bakery goods at a level of 150 milligrams of β-glucans [111]. In the European Union, the fruiting body is accepted based on its long history of traditional use. At the same time, the mycelium and its derivatives are subject to novel food regulation, ensuring their safety through controlled evaluation procedures [112]. These regulatory approvals reinforce *G. lucidum’s* reputation as a safe and valuable functional ingredient.

Clinical trials have explored the safety of *G. lucidum* in humans. For instance, a 16-week randomized, double-blind, placebo-controlled trial assessed the safety and efficacy of *G. lucidum* (3 g/day) in individuals with type 2 diabetes mellitus and metabolic syndrome. The study reported no significant adverse effects or alterations in hematological and biochemical safety markers, indicating good tolerability [113].

On the other hand, acute toxicity studies have shown that *G. lucidum* is safe, with a maximum tolerated dose exceeding 20 g/kg body weight in animal models, indicating minimal risk of toxicity, even at high doses. The recommended dose for humans is approximately 0.07 g/kg body weight. Chronic toxicity studies further support this, with prolonged administration of *G. lucidum* powder in animals showing no significant adverse effects on organ function, blood chemistry, or histological structure [114]. For example, rats given mushroom powder for long periods did not show changes in activity levels, foods, or weight [115].

The typical dosage for *G. lucidum* varies depending on its form and purpose. For powdered forms, the safe range is 1.5 to 6 g/day, while concentrated extracts are commonly consumed in doses of 150 to 300 mg/day. Liquid is usually taken in 1 to 3 mL/day. Clinical studies consistently show that these dosages are well-tolerated, with minimal side effects. Occasionally, mild gastrointestinal symptoms such as nausea or dry mouth have been reported, but these effects are transient and resolve with continued use or dose adjustments [116].

It is noteworthy that wild *G. lucidum* mushrooms contain low levels of antinutrients such as tannins, phytates, and oxalates. These compounds can interfere with nutrient absorption, but proper processing, such as soaking or drying, significantly diminishes their levels, ensuring that the mushroom’s nutritional benefits are preserved [55]. Processing methods, such as drying or boiling, have effectively lowered the levels of antinutrients, including oxalates and phytates, as reported by Akinyeye et al. [57]. Also, fermentation effectively reduces antinutrients in *G. lucidum*, improving its nutritional profile and bioavailability. During fermentation, microorganisms such as bacteria and fungi produce enzymes like phytase, tannase, and oxalase, which degrade antinutrients such as phytates, tannins, and oxalates. This enzymatic activity enhances mineral absorption by breaking down complexes that inhibit essential elements like calcium, zinc, and iron bioavailability [117,118]. Studies have shown that solid-state and submerged fermentation significantly decrease antinutrient levels while increasing the bioactivity of polysaccharides and other beneficial compounds. Thus, fermentation transforms *G. lucidum* into a more nutritionally potent product [119,120].

It is rare for mushrooms to cause allergic reactions or symptoms similar to allergies when consumed or used as dietary supplements. While isolated cases of mushroom allergies have been documented, there are no known reports of such reactions to *G. lucidum* mushrooms. *G. lucidum* extract is often employed to alleviate allergy-related symptoms and manage allergic conditions [121]. Special caution is advised for specific populations, such as pregnant or lactating women, for whom safety data are limited, and for children, as specific dosage guidelines remain poorly researched [116]. In addition, people with autoimmune conditions should use *G. lucidum* with caution, as its immune-modulating effects may interfere with immunosuppressive therapies or exacerbate symptoms [114,121,122,123].

## 6. Conclusions

*G. lucidum*, often referred to as the “mushroom of immortality”, holds significant promise as a valuable natural resource in both traditional and modern medicine. Rich in bioactive compounds, including polysaccharides, triterpenoids, and peptides, this mushroom exhibits a wide range of therapeutic properties. Notably, it has been shown to possess immune-modulating, antioxidant, anti-inflammatory, and anti-cancer effects, making it useful in the prevention and management of chronic diseases such as diabetes, cardiovascular disorders, and viral infections.

In recent years, advancements in extraction techniques, such as improved solvent methods and novel technologies, have enhanced the bioavailability of *G. lucidum* compounds, expanding their potential applications in pharmaceuticals, functional foods, and cosmetics. These advancements have opened the door for the development of more effective and targeted therapeutic products.

Despite its promising benefits, *G. lucidum* requires further research to optimize its clinical applications, refine industrial processes, and assess its long-term safety in specific populations, such as pregnant or lactating women, children, or individuals with underlying health conditions. While it is generally regarded as safe, these populations should be carefully considered in future studies to ensure the safe and appropriate use of *G. lucidum*.

Overall, *G. lucidum* presents an exciting opportunity for addressing modern health challenges, offering potential solutions for the prevention and management of various chronic conditions. Continued exploration of its bioactive compounds, clinical efficacy, and safety will further cement its role as a valuable natural therapeutic resource in both traditional and modern healthcare settings.

## References

[1] Sanodiya B.S., Thakur G.S., Baghel R.K., Prasad G.B., Bisen P.S. (2009). *Ganoderma lucidum*: A potent pharmacological macrofungus. Curr. Pharm. Biotechnol..

[2] Khatian N., Aslam M. (2018). A review of *Ganoderma lucidum* (Reishi): A miraculous medicinal mushroom. Inven. Rapid: Ethnopharmacol..

[3] Wachtel-Galor S., Yuen J., Buswell J.A., Benzie I.F. (2011). *Ganoderma lucidum* (Lingzhi or Reishi): A medicinal mushroom. Herbal Medicine: Biomolecular and Clinical Aspects.

[4] Xu C., Caserta S., Gangemi S., Pioggia G., Allegra A. (2024). Preparing *Ganoderma lucidum* slices by improved steam explosion enhances their apparent, functional and structural properties. Innov. Food Sci. Emerg. Technol..

[5] Cancemi G., Caserta S., Gangemi S., Pioggia G., Allegra A. (2024). Exploring the Therapeutic Potential of *Ganoderma lucidum* in Cancer. J. Clin. Med..

[6] Blundell R., Camilleri E., Baral B., Karpiński T.M., Neza E., Atrooz O.M. (2023). The Phytochemistry of Ganoderma Species and their Medicinal Potentials. Am. J. Chin. Med..

[7] Lau M.F., Phan C.W., Sabaratnam V., Kuppusamy U.R. (2024). Bibliometric, taxonomic, and medicinal perspectives of *Ganoderma neo-japonicum* Imazeki: A mini review. Mycology.

[8] (2024). Freepik. https://www.freepik.com/premium-photo/colorful-shelf-mushrooms_250732101.htm#fromView=search&page=1&position=48&uuid=c835a792-df01-433b-99e7-274527938045.

[9] Zhou L.-W., Cao Y., Wu S.-H., Vlasák J., Li D.-W., Li M.-J., Dai Y.-C. (2015). Global diversity of the *Ganoderma lucidum* complex (Ganodermataceae, Polyporales) inferred from morphology and multilocus phylogeny. Phytochemistry.

[10] Hennicke F., Cheikh-Ali Z., Liebisch T., Maciá-Vicente J.G., Bode H.B., Piepenbring M. (2016). Distinguishing commercially grown *Ganoderma lucidum* from *Ganoderma lingzhi* from Europe and East Asia on the basis of morphology, molecular phylogeny, and triterpenic acid profiles. Phytochemistry.

[11] He X., Chen Y., Li Z., Fang L., Chen H., Liang Z., Abozeid A., Yang Z., Yang D. (2023). Germplasm resources and secondary metabolism regulation in Reishi mushroom (*Ganoderma lucidum*). Chin. Herb. Med..

[12] Liu J., Zhang B., Wang L., Li S., Long Q., Xiao X. (2024). Bioactive components, pharmacological properties and underlying mechanism of *Ganoderma lucidum* spore oil: A review. Chin. Herb. Med..

[13] McMeekin D. (2004). The perception of *Ganoderma lucidum* in Chinese and Western culture. Mycologist.

[14] Sharma C., Bhardwaj N., Sharma A., Tuli H.S., Batra P., Beniwal V., Gupta G.K., Sharma A.K. (2019). Bioactive metabolites of *Ganoderma lucidum*: Factors, mechanism and broad spectrum therapeutic potential. J. Herb. Med..

[15] Ahmad M.F., Alsayegh A., Ahmad F.A., Akhtar M.S., Alavudeen S.S., Bantun F., Wahab S., Ahmed A., Ali M., Elbendary E.Y. (2024). *Ganoderma lucidum*: Insight into antimicrobial and antioxidant properties with development of secondary metabolites. Heliyon.

[16] Tong A., Wu W., Chen Z., Wen J., Jia R., Liu B., Cao H., Zhao C. (2023). Modulation of gut microbiota and lipid metabolism in rats fed high-fat diets by *Ganoderma lucidum* triterpenoids. Curr. Res. Food Sci..

[17] Gao Y.-Y., Zhou Y.-H., Liu X.-P., Di B., He J.-Y., Wang Y.-T., Guo P.-T., Zhang J., Wang C.-K., Jin L. (2024). *Ganoderma lucidum* polysaccharide promotes broiler health by regulating lipid metabolism, antioxidants, and intestinal microflora. Int. J. Biol. Macromol..

[18] Li M., Yu L., Zhao J., Zhang H., Chen W., Zhai Q., Tian F. (2021). Role of dietary edible mushrooms in the modulation of gut microbiota. J. Funct. Foods.

[19] Wu P., Zhang C., Yin Y., Zhang X., Li Q., Yuan L., Sun Y., Zhou S., Ying S., Wu J. (2024). Bioactivities and industrial standardization status of *Ganoderma lucidum*: A comprehensive review. Heliyon.

[20] Zheng S., Zhang W., Liu S. (2021). Optimization of ultrasonic-assisted extraction of polysaccharides and triterpenoids from the medicinal mushroom *Ganoderma lucidum* and evaluation of their in vitro antioxidant capacities. PLoS ONE.

[21] Do D.T., Lam D.H., Nguyen T., Phuong Mai T.T., Phan L.T.M., Vuong H.T., Nguyen D.V., Linh N.T.T., Hoang M.N., Mai T.P. (2021). Utilization of Response Surface Methodology in Optimization of Polysaccharides Extraction from Vietnamese Red *Ganoderma lucidum* by Ultrasound-Assisted Enzymatic Method and Examination of Bioactivities of the Extract. Sci. World J..

[22] Huang S.-Q., Ning Z.-X. (2010). Extraction of polysaccharide from *Ganoderma lucidum* and its immune enhancement activity. Int. J. Biol. Macromol..

[23] Bhadange Y.A., Carpenter J., Saharan V.K. (2024). A Comprehensive Review on Advanced Extraction Techniques for Retrieving Bioactive Components from Natural Sources. ACS Omega.

[24] Usman I., Hussain M., Imran A., Afzaal M., Saeed F., Javed M., Afzal A., Ashfaq I., Al Jbawi E., Saewan S. (2022). Traditional and innovative approaches for the extraction of bioactive compounds. Int. J. Food Prop..

[25] Chuensun T., Chewonarin T., Laopajon W., Kawee-ai A., Pinpart P., Utama-ang N. (2021). Comparative evaluation of physicochemical properties of Lingzhi (*Ganoderma lucidum*) as affected by drying conditions and extraction methods. Int. J. Food Sci. Technol..

[26] Oludemi T., Barros L., Prieto M.A., Heleno S.A., Barreiro M.F., Ferreira I.C.F.R. (2018). Extraction of triterpenoids and phenolic compounds from *Ganoderma lucidum*: Optimization study using the response surface methodology. Food Funct..

[27] Parepalli Y., Pamanji S., Singh M. (2020). *Ganoderma-lucidum*-polysaccharides-extraction-yields-and-its-biological-applications. Electron. J. Biol..

[28] Tran D.D., Pham Thi H.H., Phan V.M. (2022). Effects of Supercritical Carbon Dioxide Extraction (SC-CO2) on the Content of Triterpenoids in the Extracts from *Ganoderma lucidum*. Appl. Sci. Eng. Prog..

[29] Huang S.-Q., Li J.-W., Wang Z., Pan H.-X., Chen J.-X., Ning Z.-X. (2010). Optimization of Alkaline Extraction of Polysaccharides from *Ganoderma lucidum* and Their Effect on Immune Function in Mice. Molecules.

[30] Fesa Putra K., Siti M., Sugeng W., Wahyudiono, Motonobu G. (2021). Yield and Extraction Rate Analysis of Phytochemical Compounds from *Eucheuma cottonii*, *Ganoderma lucidum*, and *Gracilaria* sp. using Subcritical Water Extraction. ASEAN J. Chem. Eng..

[31] Kao C., Jesuthasan A.C., Bishop K.S., Glucina M.P., Ferguson L.R. (2013). Anti-cancer activities of *Ganoderma lucidum*: Active ingredients and pathways. Funct. Foods Health Dis..

[32] Azi F., Wang Z., Chen W., Lin D., Xu P. (2024). Developing *Ganoderma lucidum* as a next-generation cell factory for food and nutraceuticals. Trends Biotechnol..

[33] Zhang H., Zhang J., Liu Y., Tang C. (2023). Recent Advances in the Preparation, Structure, and Biological Activities of β-Glucan from Ganoderma Species: A Review. Foods.

[34] Chen S.-N., Nan F.H., Liu M.W., Yang M.F., Chang Y.C., Chen S. (2023). Evaluation of Immune Modulation by β-1,3; 1,6 D-Glucan Derived from *Ganoderma lucidum* in Healthy Adult Volunteers, A Randomized Controlled Trial. Foods.

[35] Gao X., Homayoonfal M. (2023). Exploring the anti-cancer potential of *Ganoderma lucidum* polysaccharides (GLPs) and their versatile role in enhancing drug delivery systems: A multifaceted approach to combat cancer. Cancer Cell Int..

[36] Chen S., Nan F.-H., Liu M.-W., Yang M.-F., Chang Y.-C., Chen S. (2016). Cytotoxic lanostane-type triterpenoids from the fruiting bodies of *Ganoderma lucidum* and their structure–activity relationships. Oncotarget.

[37] Galappaththi M.C.A., Patabendige N.M., Premarathne B.M., Hapuarachchi K.K., Tibpromma S., Dai D.Q., Suwannarach N., Rapior S., Karunarathna S.C. (2022). A Review of Ganoderma Triterpenoids and Their Bioactivities. Biomolecules.

[38] Zheng C., Rangsinth P., Shiu P.H.T., Wang W., Li R., Li J., Kwan Y.W., Leung G.P.H. (2023). A Review on the Sources, Structures, and Pharmacological Activities of Lucidenic Acids. Molecules.

[39] Xia Q., Zhang H., Sun X., Zhao H., Wu L., Zhu D., Yang G., Shao Y., Zhang X., Mao X. (2014). A Comprehensive Review of the Structure Elucidation and Biological Activity of Triterpenoids from *Ganoderma* spp.. Molecules.

[40] Dat T.D., Viet N.D., Thanh V.H., Linh N.T.T., Ngan N.T.K., Nam H.M., Phong M.T., Hieu N.H. (2022). Optimization of Triterpenoid Extraction from *Ganoderma lucidum* by Ethanol-Modified Supercritical Carbon Dioxide and the Biological Properties of the Extract. ChemistrySelect.

[41] Raza S.H.A., Zhong R., Li X., Pant S.D., Shen X., BinMowyna M.N., Luo L., Lei H. (2024). *Ganoderma lucidum* triterpenoids investigating their role in medicinal applications and genomic protection. J. Pharm. Pharmacol..

[42] Sova M., Saso L. (2020). Natural Sources, Pharmacokinetics, Biological Activities and Health Benefits of Hydroxycinnamic Acids and Their Metabolites. Nutrients.

[43] Masjedi M., Nateghi L., Berenjy S., Eshaghi M.R. (2022). Determination of Antioxidant and Antimicrobial Compounds of *Ganoderma lucidum* Extract in Laboratory Different Conditions. Chem. Methodol..

[44] Valanciene E., Jonuskiene I., Syrpas M., Augustiniene E., Matulis P., Simonavicius A., Malys N. (2020). Advances and Prospects of Phenolic Acids Production, Biorefinery and Analysis. Biomolecules.

[45] Mizzi L., Chatzitzika C., Gatt R., Valdramidis V. (2020). HPLC Analysis of Phenolic Compounds and Flavonoids with Overlapping Peaks. Food Technol. Biotechnol..

[46] Rangsinth P., Sharika R., Pattarachotanant N., Duangjan C., Wongwan C., Sillapachaiyaporn C., Nilkhet S., Wongsirojkul N., Prasansuklab A., Tencomnao T. (2023). Potential Beneficial Effects and Pharmacological Properties of Ergosterol, a Common Bioactive Compound in Edible Mushrooms. Foods.

[47] Obodai M., Mensah D.L., Fernandes Â., Kortei N.K., Dzomeku M., Teegarden M., Schwartz S.J., Barros L., Prempeh J., Takli R.K. (2017). Chemical Characterization and Antioxidant Potential of Wild Ganoderma Species from Ghana. Molecules.

[48] Papoutsis K., Grasso S., Menon A., Brunton N.P., Lyng J.G., Jacquier J.-C., Bhuyan D.J. (2020). Recovery of ergosterol and vitamin D2 from mushroom waste-Potential valorization by food and pharmaceutical industries. Trends Food Sci. Technol..

[49] Lei X., Zhi C., Huang W., Sun X., Gao W., Yin X., Zhang X., Liang C., Zhang H., Sun F. (2020). Recombinant *Ganoderma lucidum* Immunomodulatory Protein Improves the Treatment for Chemotherapy-Induced Neutropenia. Front. Pharmacol..

[50] Yeh C.-H., Chen H.-C., Yang J.-J., Chuang W.-I., Sheu F. (2010). Polysaccharides PS-G and Protein LZ-8 from Reishi (*Ganoderma lucidum*) Exhibit Diverse Functions in Regulating Murine Macrophages and T Lymphocytes. J. Agric. Food Chem..

[51] Lin H.-J., Chang Y.-S., Lin L.-H., Haung C.-F., Wu C.-Y., Ou K.-L. (2014). An Immunomodulatory Protein (Ling Zhi-8) from a *Ganoderma lucidum* Induced Acceleration of Wound Healing in Rat Liver Tissues after Monopolar Electrosurgery. Evid.-Based Complement. Altern. Med..

[52] Drzewiecka B., Wessely-Szponder J., Świeca M., Espinal P., Fusté E., Fernández-De La Cruz E. (2024). Bioactive Peptides and Other Immunomodulators of Mushroom Origin. Biomedicines.

[53] Kumar P., Kumar M., Bedi O., Gupta M., Kumar S., Jaiswal G., Rahi V., Yedke N.G., Bijalwan A., Sharma S. (2021). Role of vitamins and minerals as immunity boosters in COVID-19. Inflammopharmacology.

[54] El Sheikha A.F. (2022). Nutritional Profile and Health Benefits of *Ganoderma lucidum* “Lingzhi, Reishi, or Mannentake” as Functional Foods: Current Scenario and Future Perspectives. Foods.

[55] Ogbe A., Obeka A. (2013). Proximate, mineral and anti-nutrient composition of wild *Ganoderma lucidum*: Implication on its utilization in poultry production. Iran. J. Appl. Anim. Sci..

[56] Senila M., Senila L., Resz M.-A. (2024). Chemical composition and nutritional characteristics of popular wild edible mushroom species collected from North-Western Romania. J. Food Compos. Anal..

[57] Akinyeye R., Oluwadunsin A., Omoyeni Akinwunmi O. (2010). Proximate, Mineral, Anti-Nutrients, Phyto-Chemical Screening and Amino Acid Compositions of the Leaves of Pterocarpus Mildbraedi Harms. Electron. J. Environ. Agric. Food Chem..

[58] Muhammad A., Dangoggo S.M., Tsafe A., Adams I., Atiku F. (2011). Proximate, Minerals and Anti-nutritional Factors of *Gardenia aqualla* (*Gauden dutse*) Fruit Pulp. J. Nutr. Asian Netw. Sci. Inf..

[59] Brown H.A., Marnett L.J. (2011). Introduction to Lipid Biochemistry, Metabolism, and Signaling. Chem. Rev..

[60] Vani Raju M., Kaniyur Chandrasekaran M., Muthaiyan Ahalliya R., Velliyur Kanniappan G. (2025). Reconnoitering the role of Lipid Metabolites in Ferroptosis. Adv. Redox Res..

[61] Salvatore M.M., Elvetico A., Gallo M., Salvatore F., DellaGreca M., Naviglio D., Andolfi A. (2020). Fatty Acids from *Ganoderma lucidum* Spores: Extraction, Identification and Quantification. Appl. Sci..

[62] Kang Q., Chen S., Li S., Wang B., Liu X., Hao L., Lu J. (2019). Comparison on characterization and antioxidant activity of polysaccharides from *Ganoderma lucidum* by ultrasound and conventional extraction. Int. J. Biol. Macromol..

[63] The Nutritional Profile of Reishi Mushroom: Health Benefits + History. 2019. Peak and Valley. https://peakandvalley.co/blogs/wellness-library/the-nutritional-profile-of-reishi-mushroom-health-benefits-history?srsltid=AfmBOoq1xhpwYGoYBLEjxaUMErGdpOuWGxhaBCG2jxmi5l9qPH8mC9R7.

[64] Nosewicz J., Spaccarelli N., Roberts K.M., Hart P.A., Kaffenberger J.A., Trinidad J.C., Kaffenberger B.H. (2022). The epidemiology, impact, and diagnosis of micronutrient nutritional dermatoses. Part 2: B-complex vitamins. J. Am. Acad. Dermatol..

[65] Gharib M.A.-A., Elhassaneen Y.A.E.E., Radwan H. (2022). Nutrients and Nutraceuticals Content and In Vitro Biological Activities of Reishi Mushroom (*Ganoderma lucidum*) Fruiting Bodies. Alex. Sci. Exch. J..

[66] Saghiri M.A., Asatourian A., Ershadifar S., Moghadam M.M., Sheibani N. (2017). Vitamins and regulation of angiogenesis: [A, B1, B2, B3, B6, B9, B12, C, D, E, K]. J. Funct. Foods.

[67] Artusa P., White J.H. (2024). Vitamin D and Its Analogues in Immune System Regulation. Pharmacol. Rev..

[68] See X.Z., Yeo W.S., Saptoro A. (2024). A comprehensive review and recent advances of vitamin C: Overview, functions, sources, applications, market survey and processes. Chem. Eng. Res. Des..

[69] Eggersdorfer M., Schmidt K., Péter S., Richards J., Winklhofer-Roob B., Hahn A., Obermüller-Jevic U. (2024). Vamin E: Not only a single stereoisomer. Free Radic. Biol. Med..

[70] Kozarski M., Klaus A., Špirović Trifunović B., Miletić S., Lazić V., Žižak Ž., Vunduk J. (2023). Identifying the biological potential of Western Balkan Polypore mushroom species to mitigate the negative effects of global mushroom cultivation. Preprints.

[71] Unlu A., Nayir E., Kirca O., Ozdogan M. (2016). *Ganoderma lucidum* (Reishi Mushroom) and cancer. J. Buon.

[72] Ahmad M.F., Ahmad F.A., Khan M.I., Alsayegh A.A., Wahab S., Alam M.I., Ahmed F. (2021). *Ganoderma lucidum*: A potential source to surmount viral infections through β-glucans immunomodulatory and triterpenoids antiviral properties. Int. J. Biol. Macromol..

[73] Bettelli E., Korn T., Kuchroo V.K. (2007). Th17: The third member of the effector T cell trilogy. Curr. Opin. Immunol..

[74] Zhao R., Chen Q., He Y.-m. (2018). The effect of *Ganoderma lucidum* extract on immunological function and identify its anti-tumor immunostimulatory activity based on the biological network. Sci. Rep..

[75] Cheng S., Sliva D. (2015). *Ganoderma lucidum* for Cancer Treatment: We Are Close but Still Not There. Integr. Cancer Ther..

[76] Ma Y., Han J., Wang K., Han H., Hu Y., Li H., Wu S., Zhang L. (2024). Research progress of *Ganoderma lucidum* polysaccharide in prevention and treatment of *Atherosclerosis*. Heliyon.

[77] Meng M., Wang L., Yao Y., Lin D., Wang C., Yao J., Sun H., Liu M. (2023). *Ganoderma lucidum* polysaccharide peptide (GLPP) attenuates rheumatic arthritis in rats through inactivating NF-κB and MAPK signaling pathways. Phytomedicine.

[78] Rowaiye A., Wilfred O.I., Onuh O.A., Bur D., Oni S., Nwonu E.J., Ibeanu G., Oli A.N., Wood T.T. (2022). Modulatory Effects of Mushrooms on the Inflammatory Signaling Pathways and Pro-inflammatory Mediators. Clin. Complement. Med. Pharmacol..

[79] Hapuarachchi K., Wen T., Jeewon R., Wu X., Kang J. (2016). Mycosphere Essays 15. *Ganoderma lucidum*-are the beneficial medical properties substantiated?. Mycosphere.

[80] Adeyi A.O., Awosanya S.A., Adeyi O.E., James A.S., Adenipekun C.O. (2021). *Ganoderma lucidum* ethanol extract abrogates metabolic syndrome in rats: In vivo evaluation of hypoglycemic, hypolipidemic, hypotensive and antioxidant properties. Obes. Med..

[81] Liu Y.-J., Du J.-L., Cao L.-P., Jia R., Shen Y.-J., Zhao C.-Y., Xu P., Yin G.-J. (2015). Anti-inflammatory and hepatoprotective effects of *Ganoderma lucidum* polysaccharides on carbon tetrachloride-induced hepatocyte damage in common carp (*Cyprinus carpio* L.). Int. Immunopharmacol..

[82] Ding L., Shangguan H., Wang X., Liu J., Shi Y., Xu X., Xie Y. (2025). Extraction, purification, structural characterization, biological activity, mechanism of action and application of polysaccharides from *Ganoderma lucidum*: A review. Int. J. Biol. Macromol..

[83] Jung S., Son H., Hwang C.E., Cho K.M., Park S.W., Kim H.J. (2018). *Ganoderma lucidum* Ameliorates Non-Alcoholic Steatosis by Upregulating Energy Metabolizing Enzymes in the Liver. J. Clin. Med..

[84] Shi Y., Sun J., He H., Guo H., Zhang S. (2008). Hepatoprotective effects of *Ganoderma lucidum* peptides against d-galactosamine-induced liver injury in mice. J. Ethnopharmacol..

[85] Zhang X.-t., Ji C.-l., Fu Y.-j., Yang Y., Xu G.-y. (2024). Screening of active components of *Ganoderma lucidum* and decipher its molecular mechanism to improve learning and memory disorders. Biosci. Rep..

[86] Zhang J., Wang W., Cui X., Zhu P., Li S., Yuan S., Peng D., Peng C. (2024). *Ganoderma lucidum* ethanol extracts ameliorate hepatic fibrosis and promote the communication between metabolites and gut microbiota g_Ruminococcus through the NF-κB and TGF-β1/Smads pathways. J. Ethnopharmacol..

[87] Wang L., Zheng S., Liu Y., Ji Y., Liu X., Wang F., Li C. (2024). A nanozyme multifunctional platform based on iron doped carbon dots derived from Tibetan *Ganoderma lucidum* waste for glucose sensing, anti-counterfeiting applications, and anticancer cell effect. Talanta.

[88] Luo Y., Luo X., Xue Z., Wu M., Chen Q., Jin L. (2024). Exploring the anti-lung cancer mechanism of *Ganoderma lucidum* and its relationship with the level of immune cell infiltration based on network pharmacology and molecular docking. Oncologie.

[89] Zhong J., Fang L., Chen R., Xu J., Guo D., Guo C., Guo C., Chen J., Chen C., Wang X. (2021). Polysaccharides from sporoderm-removed spores of *Ganoderma lucidum* induce apoptosis in human gastric cancer cells via disruption of autophagic flux. Oncol. Lett..

[90] YouGuo C., ZongJi S., XiaoPing C. (2009). Modulatory effect of *Ganoderma lucidum* polysaccharides on serum antioxidant enzymes activities in ovarian cancer rats. Carbohydr. Polym..

[91] Jin H., Song C., Zhao Z., Zhou G. (2020). *Ganoderma lucidum* Polysaccharide, an Extract from *Ganoderma lucidum*, Exerts Suppressive Effect on Cervical Cancer Cell Malignancy through Mitigating Epithelial-Mesenchymal and JAK/STAT5 Signaling Pathway. Pharmacology.

[92] Nandi P., Mitra S., Mitra D.M., Paira D.M.K., Nandi D.D.K. (2023). Effect of *Ganoderma lucidum* on physiological indices and gut microflora: A review. Meas. Food.

[93] Ahmad M.F., Ahmad F.A., Hasan N., Alsayegh A.A., Hakami O., Bantun F., Tasneem S., Alamier W.M., Babalghith A.O., Aldairi A.F. (2024). *Ganoderma lucidum*: Multifaceted mechanisms to combat diabetes through polysaccharides and triterpenoids: A comprehensive review. Int. J. Biol. Macromol..

[94] Xiao C., Wu Q., Zhang J., Xie Y., Cai W., Tan J. (2017). Antidiabetic activity of *Ganoderma lucidum* polysaccharides F31 down-regulated hepatic glucose regulatory enzymes in diabetic mice. J. Ethnopharmacol..

[95] Hu L., Yu L., Cao Z., Wang Y., Zhu C., Li Y., Yin J., Ma Z., He X., Zhang Y. (2024). Integrating transcriptomics, metabolomics, and network pharmacology to investigate multi-target effects of Sporoderm-broken spores of *Ganoderma lucidum* on improving HFD-induced diabetic nephropathy rats. J. Pharm. Anal..

[96] Prasopthum A., Insawek T., Pouyfung P. (2022). Herbal medicine use in Thai patients with type 2 diabetes mellitus and its association with glycemic control: A cross-sectional evaluation. Heliyon.

[97] Ahmad M.F., Wahab S., Ahmad F.A., Ashraf S.A., Abullais S.S., Saad H.H. (2022). *Ganoderma lucidum*: A potential pleiotropic approach of ganoderic acids in health reinforcement and factors influencing their production. Fungal Biol. Rev..

[98] Tran H.-B., Yamamoto A., Matsumoto S., Ito H., Igami K., Miyazaki T., Kondo R., Shimizu K. (2014). Hypotensive Effects and Angiotensin-Converting Enzyme Inhibitory Peptides of Reishi (*Ganoderma lingzhi*) Auto-Digested Extract. Molecules.

[99] Sharif Swallah M., Bondzie-Quaye P., Wang H., Shao C.-S., Hua P., Alrasheed Bashir M., Benjamin Holman J., Sossah F.L., Huang Q. (2023). Potentialities of *Ganoderma lucidum* extracts as functional ingredients in food formulation. Food Res. Int..

[100] Ryu D.H., Cho J.Y., Sadiq N.B., Kim J.-C., Lee B., Hamayun M., Lee T.S., Kim H.S., Park S.H., Nho C.W. (2021). Optimization of antioxidant, anti-diabetic, and anti-inflammatory activities and ganoderic acid content of differentially dried *Ganoderma lucidum* using response surface methodology. Food Chem..

[101] Hafiane A., Pisaturo A., Favari E., Bortnick A.E. (2025). Atherosclerosis, calcific aortic valve disease and mitral annular calcification: Same or different?. Int. J. Cardiol..

[102] Xu Y., Zhang X., Yan X.-H., Zhang J.-L., Wang L.-Y., Xue H., Jiang G.-C., Ma X.-T., Liu X.-J. (2019). Characterization, hypolipidemic and antioxidant activities of degraded polysaccharides from *Ganoderma lucidum*. Int. J. Biol. Macromol..

[103] Arunachalam K., Sasidharan S.P., Yang X. (2022). A concise review of mushrooms antiviral and immunomodulatory properties that may combat against COVID-19. Food Chem. Adv..

[104] Cör Andrejč D., Knez Ž., Knez Marevci M. (2022). Antioxidant, antibacterial, antitumor, antifungal, antiviral, anti-inflammatory, and nevro-protective activity of *Ganoderma lucidum*: An overview. Front. Pharmacol..

[105] Gao Y., Tang W., Gao H., Chan E., Lan J., Li X., Zhou S. (2005). Antimicrobial Activity of the Medicinal Mushroom Ganoderma. Food Rev. Int..

[106] Lu W., Kong C., Cheng S., Xu X., Zhang J. (2023). Succinoglycan riclin relieves UVB-induced skin injury with anti-oxidant and anti-inflammatory properties. Int. J. Biol. Macromol..

[107] Hsiao Y., Shao Y., Wu Y., Hsu W., Cheng K., Yu C., Chou C., Hsieh C. (2023). Physicochemical properties and protective effects on UVA-induced photoaging in Hs68 cells of *Pleurotus ostreatus polysaccharides* by fractional precipitation. Int. J. Biol. Macromol..

[108] Chen H., Wu Y., Wang B., Kui M., Xu J., Ma H., Li J., Zeng J., Gao W., Chen K. (2024). Skin healthcare protection with antioxidant and anti-melanogenesis activity of polysaccharide purification from Bletilla striata. Int. J. Biol. Macromol..

[109] Anil Kumar N.V., Quispe C., Herrera-Bravo J., Herrera Belén L., Loren P., Salazar L.A., Silva V., Erdogan Orhan I., Senol Deniz F.S., Nemli E. (2023). Potential of Mushrooms Bioactive for the Treatment of Skin Diseases and Disorders. J. Food Biochem..

[110] Jiao C., Xie Y., Yun H., Liang H., He C., Jiang A., Wu Q., Yang B.B. (2020). The effect of Ganodermalucidum spore oil in early skin wound healing: Interactions of skin microbiota and inflammation. Aging Albany NY.

[111] U.S. Food and Drug Administration (2012). Beta Glucans Derived from Ganoderma lucidum Mycelium.

[112] European Commission (2025). EU Novel Food status Catalogue—Ganoderma lucidum. https://ec.europa.eu/food/food-feed-portal/screen/novel-food-catalogue/search.

[113] Klupp N.L., Kiat H., Bensoussan A., Steiner G.Z., Chang D.H. (2016). A double-blind, randomised, placebo-controlled trial of *Ganoderma lucidum* for the treatment of cardiovascular risk factors of metabolic syndrome. Sci. Rep..

[114] Xu J., Li P., Lin Z., Yang B. (2019). Researches and Application of Ganoderma Spores Powder. Ganoderma and Health: Biology, Chemistry and Industry.

[115] Lin Hua L.H., Jin LongZhe J.L., Che Cheng Lai C.C., Wang Xia W.X., Wang YuHui W.Y., Wang XinYu W.X. (2017). Toxicological safety evaluation of sporoderm-broken spore powders of organic *Ganoderma lucidum* of Changbai mountain. J. Food Saf. Qual..

[116] Ahmad R., Riaz M., Khan A., Aljamea A., Algheryafi M., Sewaket D., Alqathama A. (2021). *Ganoderma lucidum* (Reishi) an edible mushroom; a comprehensive and critical review of its nutritional, cosmeceutical, mycochemical, pharmacological, clinical, and toxicological properties. Phytother. Res..

[117] Arsov A., Tsigoriyna L., Batovska D., Armenova N., Mu W., Zhang W., Petrov K., Petrova P. (2024). Bacterial Degradation of Antinutrients in Foods: The Genomic Insight. Foods.

[118] Sharma R., Garg P., Kumar P., Bhatia S.K., Kulshrestha S. (2020). Microbial Fermentation and Its Role in Quality Improvement of Fermented Foods. Fermentation.

[119] Zhang R., Cen Q., Hu W., Chen H., Hui F., Li J., Zeng X., Qin L. (2024). Metabolite profiling, antioxidant and anti-glycemic activities of Tartary buckwheat processed by solid-state fermentation (SSF)with *Ganoderma lucidum*. Food Chem. X.

[120] Guo J., Tang C., Liu Y., Shi J., Vunduk J., Tang C., Feng J., Zhang J. (2025). Innovative submerged directed fermentation: Producing high molecular weight polysaccharides from *Ganoderma lucidum*. Food Chem..

[121] Paterson R.R.M. (2006). Ganoderma—A therapeutic fungal biofactory. Phytochemistry.

[122] Nie S., Zhang H., Li W., Xie M. (2013). Current development of polysaccharides from Ganoderma: Isolation, structure and bioactivities. Bioact. Carbohydr. Diet. Fibre.

[123] Kumar A. (2021). *Ganoderma lucidum*: A traditional chinese medicine used for curing tumors. Int. J. Pharm. Pharm. Sci..

